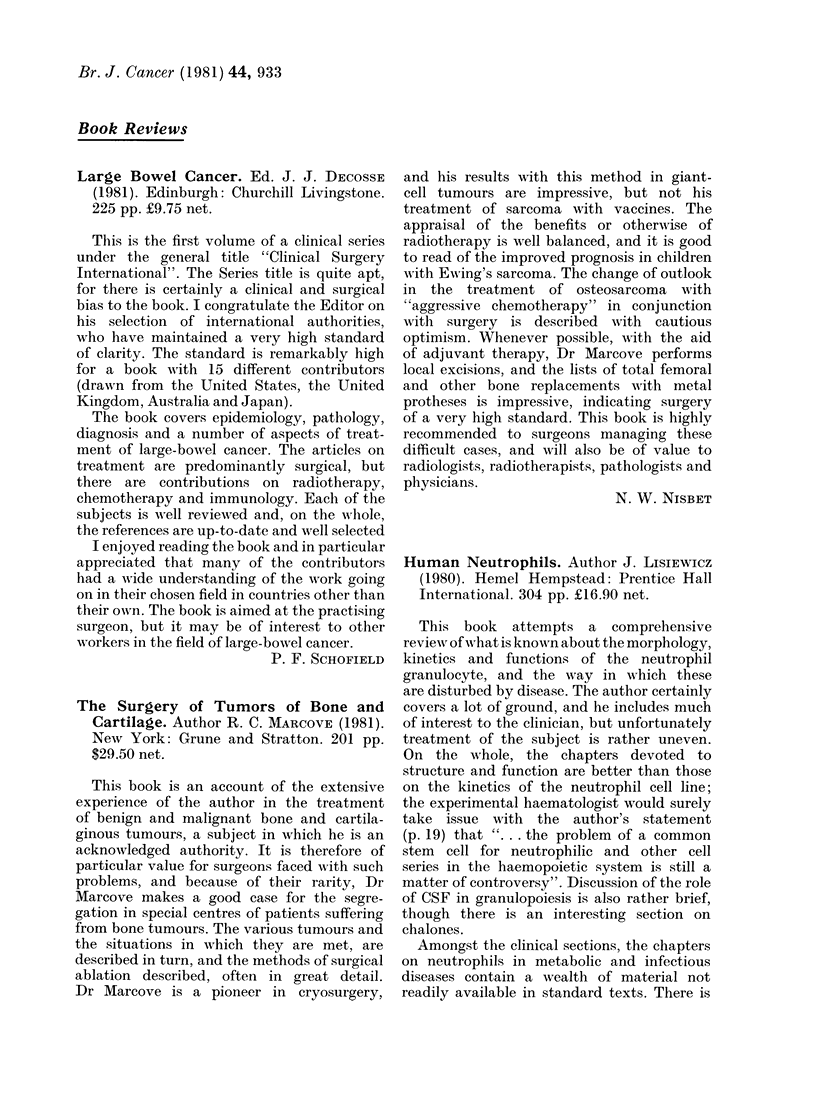# Large Bowel Cancer

**Published:** 1981-12

**Authors:** P. F. Schofield


### *Book Reviews*

**Large Bowel Cancer.** Ed. J. J. DeCosse (1981). Edinburgh: Churchill Livingstone. 225 pp. £9.75 net.

This is the first volume of a clinical series under the general title "Clinical Surgery International". The Series title is quite apt, for there is certainly a clinical and surgical bias to the book. I congratulate the Editor on his selection of international authorities, who have maintained a very high standard of clarity. The standard is remarkably high for a book with 15 different contributors (drawn from the United States, the United Kingdom, Australia and Japan).

The book covers epidemiology, pathology, diagnosis and a number of aspects of treatment of large-bowel cancer. The articles on treatment are predominantly surgical, but there are contributions on radiotherapy, chemotherapy and immunology. Each of the subjects is well reviewed and, on the whole, the references are up-to-date and well selected

I enjoyed reading the book and in particular appreciated that many of the contributors had a wide understanding of the work going on in their chosen field in countries other than their own. The book is aimed at the practising surgeon, but it may be of interest to other workers in the field of large-bowel cancer.

P. F. Schofield